# The Anti-proliferative Activity of GnRH Through Downregulation of the Akt/ERK Pathways in Pancreatic Cancer

**DOI:** 10.3389/fendo.2019.00370

**Published:** 2019-06-17

**Authors:** Linna Suo, Xiaocen Chang, Na Xu, Hongmei Ji

**Affiliations:** ^1^Department of Endocrinology and Metabolism, The Fourth Affiliated Hospital, China Medical University, Shenyang, China; ^2^Natural Sciences Department, LaGuardia Community College, City University of New York, New York City, NY, United States

**Keywords:** GnRH, proliferation, apoptosis, autophagy, pancreatic cancer

## Abstract

Gonadotropin-releasing hormone (GnRH) has been demonstrated to exert anti-proliferative functions on various tumor cells in endometrial, ovarian, bladder, or prostate cancer as a part of the autocrine system. In addition, the expression levels of GnRH and its receptor had been identified in breast cancer or non-reproductive cancers, such as glioblastoma and pancreatic cancer. Previous studies have reported abnormal GnRH expression in several malignant tumors, suggesting that GnRH and its receptor might be essential for tumourigenesis. In the present study, we attempted to clarify the mechanisms underlying GnRH function in cell proliferation in pancreatic cancer. Our results indicated that GnRH expression might be essential for the malignancy of pancreatic cancer. We then found that GnRH overexpression can induce cell apoptosis through activating the Bcl-2/Bax pathway and autophagy might be involved in the GnRH-mediated apoptosis in Panc1 cells. Further investigation showed that the inhibition of GnRH may promote tumor invasion and migration through upregulation of MMP2 expression in pancreatic cancer cells. Moreover, our results indicated that GnRH can regulate the Akt/ERK1/2 pathways to promote cell proliferation by inhibiting cell apoptosis in Panc1 cells. Therefore, our finding exhibited that the regulation of GnRH expression may be essential for tumourigenesis in pancreatic cancer, and might be a potential target for the treatment of the patients with pancreatic cancer.

## Introduction

Pancreatic cancer is a rare, but lethal malignant tumor, which is the third most deadly cancer, and there were over 50,000 estimated cases and 43,090 deaths in the United States (1). The treatment and the current 5-year survival rate (~8%) of pancreatic cancer have no progression in recent years (1). Its poor prognosis is usually due to the delayed diagnosis, which resulted in most of patients with pancreatic cancer were diagnosed at the advanced stages (III or IV stage) (2). Thus, the identification of the potential biomarkers responsible for the development of pancreatic cancer may be essential for the diagnosis and improvements in the treatment of the patients with pancreatic cancer.

Gonadotropin-releasing hormone (GnRH) is produced in the small subset of neurons of the septal-preoptic-hypothalamic region (3, 4). These neurons secrete the neurohormone into the hypophyseal portal circulation, through which it reaches the anterior pituitary to stimulate the synthesis/release of these two gonadotropins that in turn regulate gonadal gender steroid production (5). In addition, several studies have indicated that cancers of the ovary, endometrium, and breast have receptors for GnRH (6), suggesting that the GnRH expression may be related to tumor progression. Previous reports have also indicated that GnRH and its receptor are present in endometrial and ovarian cancer specimens and cell lines (7). Kakar et al. revealed that the DNA sequences of GnRH receptors (GnRHR) in ovarian and breast cancers are identical to those within the pituitary (8). Harris et al. showed that the gene expression of GnRH in human breast cancer cell lines (9). Moreover, the high-expression of GnRH and its receptor had been found in several cancers from non-reproductive tissues, including the urinary bladder cancer, glioblastoma, lung cancer, and breast cancer (10–12). Furthermore, a recent study indicated that GnRH agonists have strong anti-tumor activity, which can reduce cell proliferation in ovarian, endometrial, and breast cancer cells (13). A GnRH antagonist can cause the reduction of cell proliferation in a dose- and time-dependent manner in various tumors (13–16). Therefore, all this evidence indicates that GnRH may play an important role as a modulator of tumor growth in various malignant tumors, which might provide potential targets for therapy with GnRH analogs.

Many reports have investigated the functions of agonists/antagonists of GnRH in malignant tumors. However, fewer studies have focused on the effects of autocrine/paracrine GnRH on the progression of malignant tumors. In this study, investigated the functions of autocrine/paracrine GnRH in the progression in pancreatic cancer. Our results showed that GnRH expression might be involved in tumor malignancy in patients with pancreatic cancer. In addition, we found the inhibition of GnRH expression can promote proliferation by inhibiting autophagy and apoptosis in pancreatic cancer cells. Moreover, our results showed that GnRH expression can regulate tumor metastasis in pancreatic cancer. Further study revealed that Akt/ERK signaling pathways are involved in this process in pancreatic cancer cells. These findings provide insight into the mechanism by which GnRH contributes to tumor progression and metastasis, which may improve anti-tumor treatment of pancreatic cancer.

## Materials and Methods

### Cell Culture and Treatments

Pancreatic cancer Panc1 cells were obtained from American Type Culture Collection (ATCC; Manassas, VA, USA), the cells were maintained in DMEM (Gibco, Thermo Fisher Scientific, Waltham, MA, USA) supplemented with 10% fetal bovine serum (FBS; Gibco), 50 μg/ml penicillin and 100 μg/ml streptomycin (Invitrogen, Thermo Fisher Scientific) in a 5% CO_2_ humidified atmosphere at 37°C.

Chloroquine (CQ), a lysosomal inhibitor, was purchased from the Sigma Aldrich (St. Louis, MO, USA). 3-methyladenine (3-MA), a PI3K inhibitor, which can also specific inhibit autophagy, was obtained from MedChemExpress (Monmouth Junction, NJ, USA). The cells were treated with CQ at 40 μM for 2 h, or 3-MA for 24 h, according to the previous report (17). MK-2206 or SCH772984 (Selleck Company, Houston, TX, USA), specific inhibitors of the Akt or ERK1/2 signaling pathways, respectively, were added to the tissue culture medium. The final concentrations were 5 μM (MK-2206) or 10 μM (SCH772984) for treatment of Panc1 cells. Untreated cells were used as a control.

### Overexpression and Knockdown of GnRH Expression

The GnRH Crispr Activation Plasmids system, including GnRH Crispr Activation Plasmid, the Crispr/dCas9-VP64-Blast plasmid, and MS2-P65-HSF1-Hygro plasmid (sc-401425-ACT, Santa Cruz Biotechnology, Santa Cruz, CA, USA), and the GnRH HDR Plasmid (h2) system, including GnRH HDR Plasmid (h2), and GnRH Crispr/Cas9 KO plasmid (sc-401425-HDR-2, Santa Cruz Biotechnology), were used to overexpress or knockdown GnRH expression in pancreatic cancer cells, respectively. The GnRHR Crispr/Cas9 KO plasmid (sc-401783, Santa Cruz Biotechnology) was used to knockdown GnRH receptor expression in pancreatic cancer cells. Briefly, to establish stable overexpression or knockdown of GnRH (or GnRHR) protein in Panc1 cells, 1 ×10^6^ Panc1 cells were seeded in a 6-well plate. Lipofectamine 3000 (5 μl, Invitrogen) and 5 μg total plasmids were diluted in a 200 μl Opti-MEM medium (Invitrogen) and incubated for 20 min. The mixture was then added the cell culture medium and incubated with the cells for 48 h.

### Immunohistochemistry

A paraffin-embedded tissue microarray (TMA) that contained 9 cases of normal pancreatic tissues and 60 cases of pancreatic cancer specimens, which was purchased from Alenabio company (PA2072; Xi'an, China). The TMA was dewaxed with xylene, rehydrated in descending concentrations of ethanol, and then incubated with mouse anti-GnRH antibody (1:100; sc32292, Santa Cruz Biotechnology). After four-time PBST washing, the slide was then developed and counterstained with haematoxylin by using Super Sensitive Polymer HRP Detection System/DAB Kit protocol (Thermo Fisher Scientific).

### Western Blot Assay

Cells protein lysates were separated on a 10% SDS-PAGE gel and transferred to a polyvinylidene difluoride membrane (PVDF; Millipore, Billerica, MA, USA). The signals were visualized by using a SuperSignal West Pico Substrate Kit (Pierce, Thermo Fisher Scientific), and the signal fluorescence intensity was measured with ImageJ software (NIH, Bethesda, MD, USA). The primary and secondary antibodies for western blotting were as follows: rabbit anti-GnRH antibody (1:2,000; 26950-1), rabbit anti-GNRHR antibody (1:2,000; 19950-1), rabbit anti-c-myc antibody (1:2,000; 10828-1), rabbit anti-Bcl-2 antibody (1:2,000; 12789-1), rabbit anti-caspase-3 antibody (1:2,000; 19677-1), rabbit anti-caspase-9 antibody (1:2,000; 10380-1), mouse anti-p-Akt antibody (1:2,000; 66444-1), rabbit anti-Akt antibody (1:2,000; 10176-2), rabbit anti-MMP2 antibody (1:2,000; 10373-2), rabbit anti-MMP9 antibody (1:2,000; 10375-2), and rabbit anti-Beclin-1 antibody (1:2,000; 11306-1), which were obtained from ProteinTech Group Inc. (Rosemont, IL, USA); rabbit anti-LC3B antibody (1:2,000; ab192890), rabbit anti-c-myc (phosphor S62) antibody (1:2,000; ab185656), and mouse anti-β-actin antibody (1:5,000; ab8226) were obtained from Abcam company (Cambridge, UK); mouse anti-Bax antibody (1:2,000; sc-20067), mouse anti-ERK1/2 antibody (1:2,000; sc-514302), and mouse anti-p-ERK1/2 antibody (1:2,000; sc-81492) were purchased from Santa Cruz Biotechnology; and anti-GAPDH antibody (1:5,000; 100242) purchased from Sino Biological Inc. (Beijing, China). All secondary antibodies were from obtained from Jackson ImmunoResearch Laboratories Inc. (1:5000; West Grove, PA, USA).

### Cell Proliferation Assay

Cell Counting Kit-8 (CCK-8) (Beyotime, Beijing, China) was used to detect the cell proliferation in Panc1 cells. In brief, all cells were seeded at 2,000 cells/well in phenol red-free cell culture medium with 10% FBS in a 96-well-plate. Then, 10 μl CCK-8 working solution was added into each well at different time points (1, 2, 3, 4, and 5 days), and then incubated for 2 h at 37°C. And their absorbances were finally measured at 450 nm.

### Colony Formation Assay

Briefly, 5 ×10^2^ cells were seeded in a 24-well plate and then cultured for 3 weeks, with fresh medium replacement every 3 days. Cells were stained with crystal violet for 10 min and detained with PBS three times. Colonies in each well were counted using ImageJ software, and triplicate samples were prepared for each condition.

### TUNEL Assay

Cell apoptosis was detected with a One Step TUNEL Apoptosis Assay Kit (Beyotime). In brief, cells were seeded in a 24-well-plate. After three washes, the cells were fixed with 4% paraformaldehyde (PFA) for 30 min, treated with 200 μl PBST (1% Tween-20 in PBS buffer) at room temperature for 5 min and then after another two washes with PBS were treated with 50 μl TUNEL detection solution at 37°C for 1 h.

### Wound Healing Assay

All the cells were incubated in DMEM with 10% FBS, and wounded by using a 200-μl pipette tip in a 6-well plate. The wound width was photographed at different post-scratch time points (12, 24, and 36 h) under a phase-contrast microscope.

### Transwell Assay

A 24-well Corning transwell microplates (Corning Incorporated, NY, USA) was coated with 10 μl Matrigel (BD Bioscience). 2 ×10^3^ cells were seeded into the top chamber in serum-free DMEM medium. The bottom chambers were filled with DMEM medium supplemented with 10% FBS. After 24 h incubation, the cells on the upper surface were removed with a cotton swab, and the membranes were then fixed and stained with crystal violet. The invasive cells on the membranes were finally counted under a phase-contrast microscope. Each experiment was examined with five replicates.

### Statistical Analysis

The results are expressed as the means ± standard error, and differences between the means were analyzed via one-way or two-way analysis of variance (ANOVA). *p* <0.05 was regarded as statistically significance. Statistical analysis was performed using SPSS statistical software (SPSS Inc., Chicago, IL, USA).

## Results

### The Abnormal GnRH Expression in Advanced Human Pancreatic Cancer

**Figure 1:**
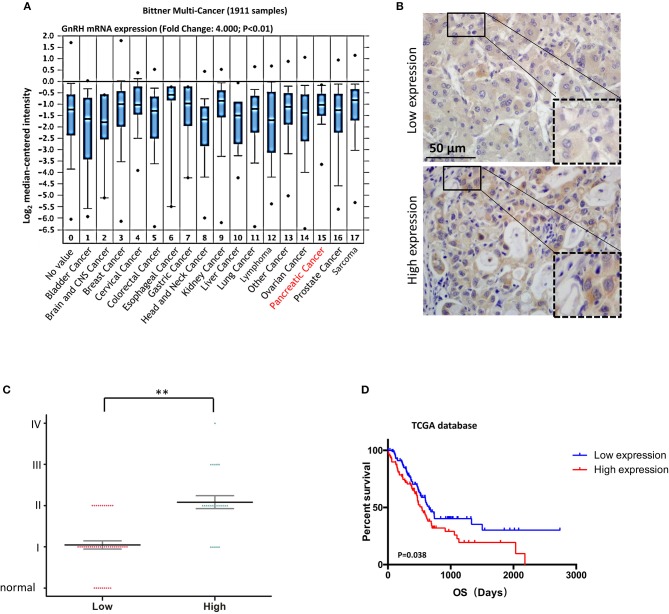
Correlation between GnRH expression and malignancy in pancreatic cancer. **(A)** Oncomine data analysis of GnRH mRNA levels in 16 types of cancer from the Ramaswamy multi-cancer datasets; **(B)** Representative images of GnRH expression in pancreatic cancer; **(C)** Pathological analysis of the correlation between GnRH expression and malignancy in pancreatic cancer. **(D)** Overall survival rates according to data from the TCGA database. ^**^p <0.01; Scale bars, 50 μm.

Since previous studies indicated that GnRH and its receptor were expressed in various malignant tumors (10, 11, 13), we expected that GnRH expression might be associated with malignancy in pancreatic cancer. Based on the Bittner multi-cancer dataset, GnRH and GnRHR were upregulated in pancreatic cancer (Figure 1A and Supplementary Figure 1). We therefore investigated the expression levels of GnRH in different stages in human pancreatic cancer. We first performed IHC for examining GnRH expression in a commercial microarray, including 9 normal/adjacent pancreatic tissues and 60 human pancreatic cancer specimens (Table 1). After analyzing the overall staining intensity, we found that GnRH immunostaining was very weak in normal and early-stage pancreatic cancer specimens (I and II), whereas the high-expression of GnRH was observed in the advanced pancreatic cancer specimens (II, III, and IV), suggesting that GnRH expression might be related to the malignancy in pancreatic cancer (Figure 1B). Further quantitative analysis revealed that the increasing GnRH expression was proportional to the malignancy of pancreatic cancer tissues and thus might have functional relevance (Figure 1C). Moreover, prognostic analysis demonstrated that the higher expression level of GnRH is positively correlated with the prognosis in the patients with pancreatic cancer in TCGA database (Figure 1D). All these evidences indicated that regulation of GnRH expression may be a potential diagnostic biomarker of for the patients with pancreatic cancer.

**Table 1:** Characteristics of patients with pancreatic cancer.

**Characteristic**	**Adenocarcinoma (*n* = 60)**	**Normal tissues (*n* = 9)**
**Age (Years)**		
≥60	23 (38%)	0
<60	37 (62%)	9
**Gender**		
Male	36 (60%)	5
Female	24 (40%)	4
**Grade**		
1–2	37 (62%)	–
3–4	23 (38%)	–
**Stage**		
I	30 (50%)	–
II	24 (40%)	–
III+IV	6 (10%)	–

### GnRH Is Involved in Cell Proliferation in Pancreatic Cancer

**Figure 2:**
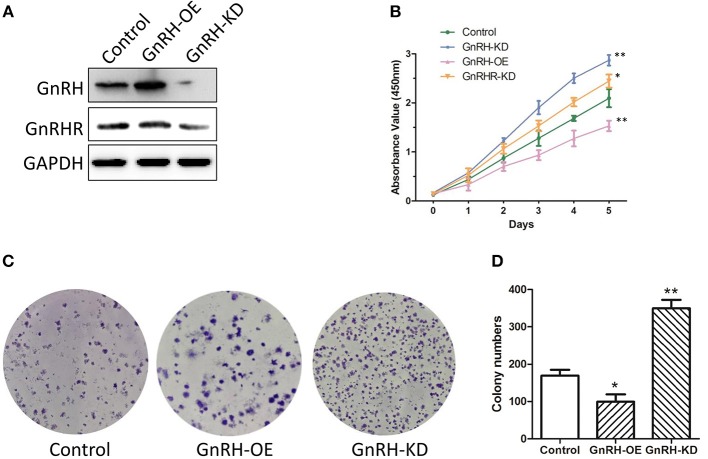
GnRH regulates pancreatic cancer cell proliferation. **(A)** the expression levels of GnRH and GnRHR in GnRH-OE, GnRH-KD, or Control group Panc1 cells; **(B)** Proliferation of GnRH-OE, GnRH-KD, GnRHR-KD, or Control group Panc1 cells; **(C)** Representative images of colony formation in GnRH-OE, GnRH-KD, or Control group Panc1 cells; **(D)** Statistical analysis of colony formation in GnRH-OE, GnRH-KD, or Control group Panc1 cells. ^*^p <0.05, ^**^p <0.01, compared with the control.

Since the expression level of GnRH was associated with the malignancy of pancreatic cancer, we predicted that GnRH might be associated with cell proliferation in pancreatic cancer. To confirm our hypothesis, we first overexpressed or inhibited GnRH expression in Panc1 cells and then examined cell proliferation in three groups: GnRH-overexpressing (GnRH-OE), GnRH-silencing (GnRH-KD), and non-treated (Control) Panc1 cells (Figure 2A). Cell proliferation assays showed that GnRH overexpression significantly inhibited cell proliferation compared with the GnRH-KD and control groups, whereas the cell proliferation of GnRHR-silencing group (GnRHR-KD) was increased in Panc1 cells, comparing with the control group (Figure 2B). Furthermore, colony formation assays indicated that GnRH-overexpressing Panc1 cells formed far fewer colonies than the GnRH-inhibited or control cells (Figures 2C,D), indicating that GnRH expression has an anti-proliferation affect in pancreatic cancer.

### GnRH Can Induce Autophagy-Related Apoptosis Through the Bcl-2/Bax Pathway in Pancreatic Cancer

**Figure 3:**
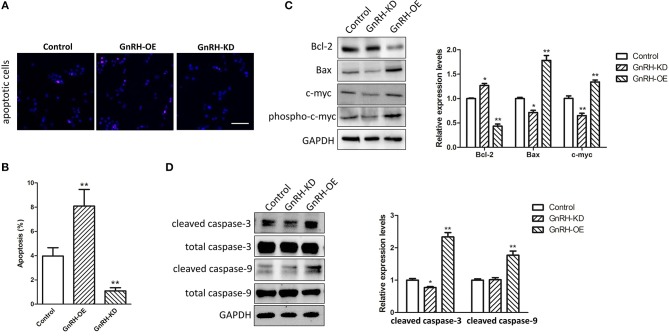
GnRH treatment inhibits cell proliferation by inducing apoptosis in pancreatic cancer cells. **(A)** TUNEL assays were performed to determine the number of apoptotic Panc1 cells in the GnRH-OE, GnRH-KD, and Control groups; **(B)** Percentage of apoptotic Panc1 cells in the GnRH-OE, GnRH-KD, and Control groups; **(C,D)** Expression levels of Bcl-2, Bax, c-myc, phosphor-c-myc, cleaved caspase-3, and cleaved caspase-9 in GnRH-OE, GnRH-KD, and Control group Panc1 cells. ^*^p <0.05, ^**^p <0.01, compared with the control; Scale bars, 100 μm.

As we known, apoptosis plays an important role in regulation of cell proliferation in various malignant tumors. We next expected that regulation of GnRH expression might promote cell proliferation by inhibiting apoptosis in pancreatic cancer cells. To investigate the mechanism by which GnRH functions in apoptosis of pancreatic cancer cells, we performed TUNEL assays to confirm whether overexpression or inhibition of GnRH expression was involved in apoptotic induction in pancreatic cancer cells. We found more apoptotic cells in GnRH-OE group Panc1 cells, whereas less apoptotic cells in GnRH-KD group Panc1 cells, suggesting that GnRH overexpression might induce apoptosis in pancreatic cancer cells (Figures 3A,B). To further investigate the possible functions of GnRH in cell apoptosis, we then examined the expression levels of Bcl-2, Bax, c-myc, phosphor-c-myc, cleaved caspase-3, and cleaved caspase-9 proteins, which are key factors in cell apoptosis (18, 19). Our results indicated that the increasing GnRH expression significantly induced downregulation of Bcl-2 expression, and upregulation of Bax, c-myc, cleaved caspase-3, and cleaved caspase-9 in Panc1 cell groups (Figures 3C,D).

**Figure 4:**
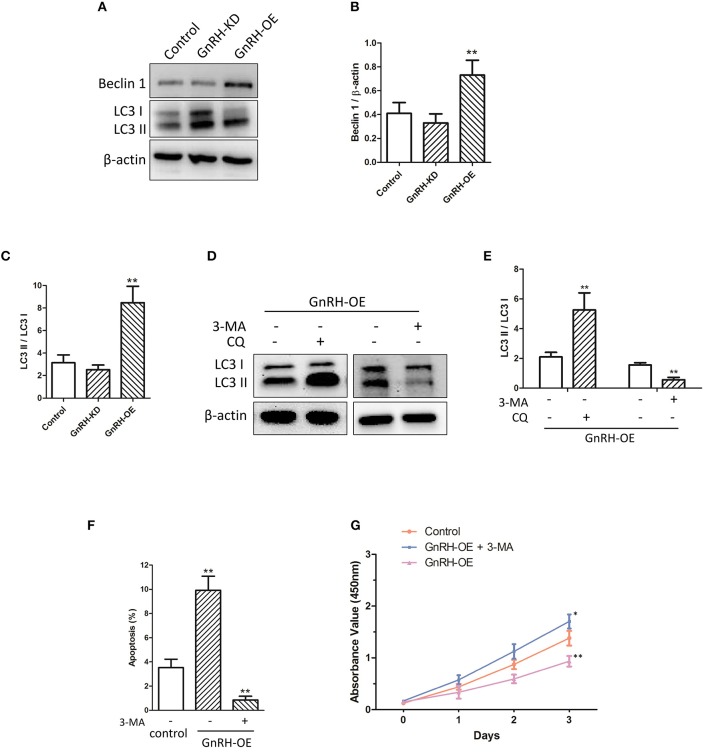
Autophagy is involved in GnRH-mediated apoptosis in pancreatic cancer cells. **(A)** The expression of Beclin 1, LC3B-I, and LC3B-II was detected via western blot analysis in GnRH-OE, GnRH-KD, and Control group Panc1 cells. Quantitative analysis of the protein Beclin 1 and β-actin expression ratio **(B)** and LC3-II and LC3-I expression ratio **(C)** in GnRH-OE, GnRH-KD, and Control group Panc1 cells. ^*^p <0.01, compared with the control. **(D,E)** LC3 II expression was regulated in CQ- or 3-MA-treated GnRH-OE Panc1 cells. **(F)** TUNEL assay revealed that 3-MA treatment inhibits apoptosis in GnRH-OE Panc1 cells. **(G)** Proliferation of GnRH-OE, 3-MA-treated GnRH-OE, or Control group Panc1 cells ^*^p <0.05, ^**^p <0.01, compared with the control.

To further investigate whether autophagy is involved in GnRH-induced apoptosis in pancreatic cancer cells, we next attempted to confirm whether overexpression of GnRH can induce autophagy-related apoptosis. Our results demonstrated that Beclin 1 expression and conversion of the cytosolic form of LC3B-I to its lipidated membrane-bound form LC3B-II were increased in the Panc1 cell GnRH-OE group (Figures 4A–C), suggesting that autophagy may be involved in GnRH-mediated apoptosis in pancreatic cancer cells. To investigate the induction of the autophagic flux in this process, GnRH-OE cells were treated with or without CQ (40 μM for 2 h) or 3MA (5 mM for 24 h), respectively. It was observed that LC3 II levels were increased in CQ-treated GnRH-OE cells, whereas decreased in 3-MA-treated GnRH-OE cells (Figures 4D,E). Furthermore, the apoptosis and cell proliferation were found to be regulated in 3-MA-treated GnRH-OE Panc1 cells (Figures 4F,G), suggesting that autophagy-related apoptosis might be involved, at least partially, in the anti-proliferative activity of GnRH in pancreatic cancer cells.

### GnRH Regulates Tumor Invasion and Migration by Inhibiting MMP2 Expression in Pancreatic Cancer Cells

**Figure 5:**
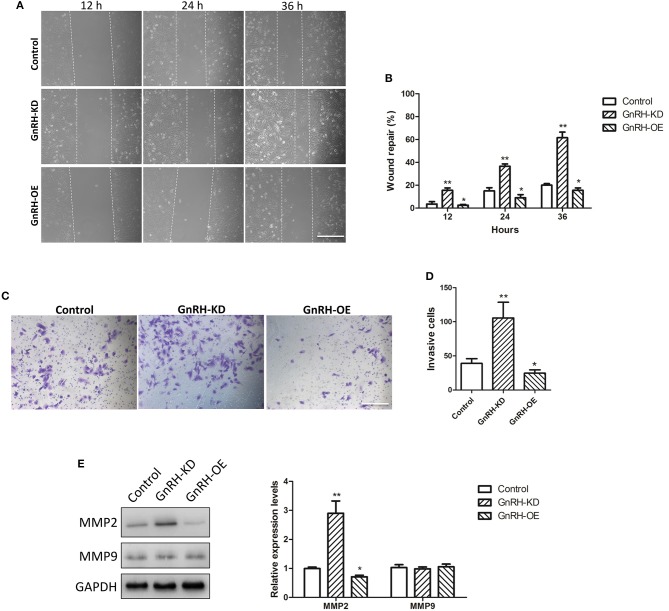
Effects of GnRH on tumor invasion and migration in pancreatic cancer cells. **(A,B)** The migration of GnRH-OE, GnRH-KD, or Control group Panc1 cells at 0, 12, 24, and 36 h; **(C,D)** Cell invasion was determined by transwell assays in GnRH-OE, GnRH-KD, and Control group Panc1 cells at 24 h. **(E)** MMP2 and MMP9 protein expression was determined in GnRH-OE, GnRH-KD, and Control group Panc1 cells. ^*^p <0.05, ^**^p <0.01, compared with the control. Scale bar, 200 μm.

Previous studies indicated that treatment with GnRH analogs can reduce the ability of cells to invade through the basement membrane and migrate in response to a cellular stimulus, and GnRH analogs also exhibited comparable anti-metastatic effects in prostate cancer cells (20, 21). Therefore, we examined whether overexpression or inhibition of GnRH was associated with tumor invasion and migration in GnRH-OE, GnRH-KD, or Control group Panc1 cells. Wound healing assays showed that inhibition of GnRH expression induced cells to migrate into the scratched area more rapidly than GnRH-overexpressing or non-treated Panc1 cells at 12, 24, or 36 h (Figures 5A,B). Similarly, further transwell assays demonstrated that inhibition of GnRH led to a higher invasive capacity in Panc1 cells (Figures 5C,D).

MMP2 and MMP9 are closely involved in tumor invasion and migration in many malignant tumors (22). To further investigate the functions of GnRH in tumor invasion and migration of pancreatic cancer cells, we examined the expression levels of MMP2 and MMP9 proteins in GnRH-OE, GnRH-KD, and Control group Panc1 cells. Notably, western blot analysis indicated upregulation of MMP2 expression in GnRH-inhibited Panc1 cells, whereas the regulation of MMP9 expression was not obvious (Figure 5E), suggesting that MMP2 might play a role in GnRH-related invasion and migration in pancreatic cancer cells.

### The Akt/ERK Pathways Are Associated With GnRH Cell Proliferation Inhibition Through Apoptosis Induction in Pancreatic Cancer Cells

**Figure 6:**
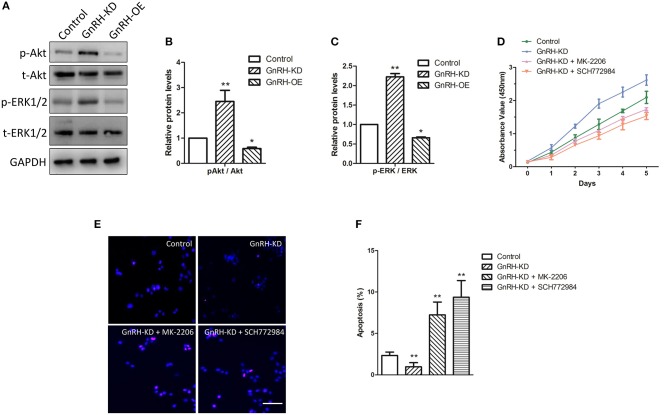
The Akt/ERK pathways are associated with GnRH-mediated cell proliferation through apoptosis induction in pancreatic cancer cells. **(A)** Expression levels of p-Akt, t-Akt, p-ERK1/2, and t-ERK1/2 were determined by western blotting in GnRH-OE, GnRH-KD, and Control group Panc1 cells; Quantitative analysis of the protein p-Akt and t-Akt expression ratio **(B)** and p-ERK1/2 and t-ERK1/2 expression ratio **(C)** in GnRH-OE, GnRH-KD, and Control group Panc1 cells; **(D)** Proliferation of GnRH-inhibited Panc1 cells with or without MK-2206 or SCH772984 treatment; Representative images **(E)** and quantitative analysis **(F)** of apoptosis of GnRH-inhibited Panc1 cells with or without MK-2206 or SCH772984 treatment. p-Akt, phosphorylated-Akt; t-Akt, total-Akt; p-ERK1/2, phosphorylated-ERK1/2; t-ERK1/2, total-ERK1/2. ^*^p <0.05, ^**^p <0.01, compared with the control. Scale bars, 100 μm.

Bcl-2 and Bax are key regulators in cell apoptosis, which are also down-stream factors of Akt and ERK pathways (23, 24). GnRH agonists were reported to inhibit mitogen-activated protein kinase (MAPK, ERK) activity (25). Additionally, a previous study indicated that GnRH can activate the PI3K/Akt pathway in pituitary gonadotropes (26), encouraging us to clarify the mechanism of GnRH-regulated cell proliferation in pancreatic cancer cells. We therefore examined activation of Akt/ERK pathways via western blot analysis. The results indicated that overexpression of GnRH significantly inhibited the level of phosphorylated Akt and ERK1/2 proteins (Figure 6A), whereas the total expression levels of Akt and ERK1/2 were not changed in GnRH-OE, GnRH-KD, or Control Panc1 cells, indicating that activation of either the Akt or ERK pathway is involved in GnRH regulation of Panc1 cell proliferation (Figures 6B,C).

To further confirm our hypothesis, rescue assays were performed, and cell proliferation and apoptosis were examined. We first detected the proliferation of GnRH-inhibited Panc1 cells treated with or without MK-2206, an inhibitor of the Akt signaling pathway. Interestingly, the treatment of MK-2206 significantly suppressed cell proliferation and promoted cell apoptosis in GnRH-inhibited Panc1 cells (Figures 6D–F). Similarly, treatment with SCH772984, a specific ERK1/2 inhibitor, also inhibited cell proliferation by promoting cell apoptosis in GnRH-inhibited Panc1 cells (Figures 6D–F). Therefore, these findings suggest that inhibition of GnRH may activate the Akt/ERK pathways to promote cell proliferation by inhibiting autophagy-related apoptosis in pancreatic cancer cells.

## Discussion

Like many malignant tumors, pancreatic cancer is hard to diagnose at its early stages, which usually found to be metastatic at the time of initial diagnosis. Currently, the surgical resection is the only curative treatment of pancreatic cancer, but only <20% patients are candidates for pancreatectomy. Moreover, the 5-year survival rate after pancreaticoduodenectomy is ~21% for negative margin resections and 11% for microscopically positive margin resections (27). Thus, a thorough understanding of the tumourigenesis process in pancreatic cancer and the identification of the effective biomarkers will be helpful for improving the diagnosis of pancreatic cancer.

Many previous studies had indicated that abnormal expression of GnRH and its receptor is found in various malignant tumors, not only in a reproductive system tumors but also in non-reproductive tissues, including breast cancer, ovarian, endometrial, prostate cancer, urinary bladder cancer, pancreatic cancer, and glioblastoma, suggesting that GnRH and its receptor might be used for targeted therapy with improved anti-tumor effects (10, 11, 28). The cytotoxic GnRH agonist Zoptarelin Doxorubicin (ZEXS-106, AN-152) was more potent than doxorubicin in the inhibition of *in vitro* cell growth in many GnRH receptor-positive cancer cell lines (28, 29). The previous study also showed that Zoptarelin Doxorubicin is a potential therapeutic option for the treatment of triple negative breast cancer, which exhibits a higher percentage of GnRH receptor-positive tumors (30). In the present study, our results indicated the abnormal expression of GnRH in advanced pancreatic cancer specimens (Figure 1), and the overexpression and inhibition of GnRH was highly involved in the proliferation in Panc1 cells (Figure 2), suggesting that GnRH might play an important role in tumourigenesis in patients with pancreatic cancer.

The expression levels of GnRH and its receptor had been demonstrated as a part of the regulatory system of cell proliferation in various human malignant tumors (10, 28). There were several reports indicated dose-dependent anti-proliferative effects of GnRH agonists in many cancer cell lines. GnRH antagonists caused anti-proliferative effects in most endometrial cancer, breast cancer, and ovarian cancer cell lines, suggesting that the dichotomy of GnRH agonists and antagonists might not apply to the GnRH system in many tumors (6, 13). It is well-known that cell proliferation is usually related to cell apoptosis in many malignant tumors (31, 32). Previous studies have also demonstrated that antagonists of GnRH-II, a version of GnRH with an alternative structural that is completely structurally conserved from fish to mammals, and different from GnRH in three amino acids, can induce apoptosis in endometrial cancer, breast cancer, and ovarian cancer cell lines through activating the intrinsic apoptosis pathway (33, 34). In addition, Zoptarelin Doxorubicin treatment can result in apoptosis in GnRH receptor-positive MiaPaCa-2 and Panc1 human pancreatic cancer cells (28), which is similar to our finding that GnRH overexpression can induce apoptosis in Panc1 cells (Figures 3A,B). Our results also indicated that the regulation of GnRH expression was associated with activation of the Bcl-2/Bax/caspase pathway in Panc1 cells (Figures 3C,D). Bcl-2 and Bax are key apoptotic factors involved in the cell apoptosis and autophagy processes (35, 36). Specifically, caspase-3/-9 are the key effector enzyme in the apoptotic processes (both intrinsic and extrinsic). In addition, the JNK-Bcl-2/Bcl-xL-Bax/Bak pathways were found to mediate crosstalk between matrine-induced autophagy and apoptosis via interplay with Beclin 1 (37). Li et al. showed that rapamycin can induce autophagy in the melanoma cell line M14 via regulation of the expression levels of Bcl-2 and Bax (38). Therefore, we expected that regulation of GnRH might be involved in cell proliferation through induction of Bcl-2/Bax-mediated autophagy-related apoptosis in pancreatic cancer cells.

GnRH is known as a regulator in different intracellular signaling cascades, including MAPK (p38/MAPK, ERK1/2, or JNK), phosphatidylinositol-3-kinase (PI3K), and phosphotyrosine phosphatase (PTP) pathways (39–42). Our results demonstrated that inhibition of GnRH was associated with the activation of either the ERK1/2 or Akt pathway in pancreatic cancer cells (Figures 6A–C). In contrast, treatment with an inhibitor of the Akt or ERK pathway significantly affected cell proliferation and apoptosis in GnRH-inhibited pancreatic cancer cells (Figures 6D–F). Several studies had revealed that the Akt/ERK pathways are tightly related to cell proliferation through apoptosis regulation in various malignant tumors. Wang et al. reported that Stachydrine hydrochloride can inhibit cell proliferation through inducing apoptosis of breast cancer cells via the inactivation of Akt and ERK pathways (43, 44). A previous study also indicated that Lupeol can inhibit proliferation and induce apoptosis of human pancreatic cancer PCNA-1 cells through the Akt/ERK pathways (44). Additionally, several previous studies indicated that the regulation of Akt/ERK pathways were associated with autophagy in various malignant tumors. Zhang et al. found that PI3K/Akt/ERK pathways can participate in mollugin-induced autophagy and apoptosis (45). The regulation of PI3K/Akt/mTOR and MEK/ERK pathways can lead to the activation of autophagy in HeLa cells (46). In contrast, Ba et al. demonstrated that allicin attenuates pathological cardiac hypertrophy by inhibiting autophagy via activation of PI3K/Akt/mTOR/ERK signaling pathway (47). We therefore expected that regulation of autocrine/paracrine GnRH expression could activate the Akt/ERK pathways, thus inhibiting cell proliferation by inducing cell apoptosis and autophagy in pancreatic cancer cells.

Notably, our results also showed that inhibition of GnRH can significantly increase the ability of Panc1 cells to invade through the basement membrane and migrate (Figure 5). Activation of the Akt/ERK pathways is often involved in tumor invasion and migration in many malignant tumors (48, 49). Furthermore, the Akt/ERK pathways can regulate the process of epithelial-mesenchymal transition (EMT) in various tumors, including glioblastoma, hepatocellular carcinoma, and colorectal cancer (50–52). Our further results indicated that GnRH can regulate the expression level of MMP2 but not MMP9 in pancreatic cancer cells (Figure 5E). Cheung et al. showed that JNK might be involved in the GnRH-stimulated invasive behavior of ovarian cancer cells by upregulating the expression of MMP2 and MMP9 (53), suggesting that the JNK signaling pathway might be involved in this process in pancreatic cancer cells. The precise mechanisms and pathways by which GnRH participates in tumor invasion and migration in pancreatic cancer require further investigation.

In summary, the present data provide evidence indicating that GnRH mediates activation of the Akt/ERK pathways, thus affecting cell proliferation, apoptosis, autophagy, invasion and migration in pancreatic cancer cells. Our findings revealed that GnRH may be a potential target for the diagnosis of patients with pancreatic cancer.

## Data Availability

The raw data supporting the conclusions of this manuscript will be made available by the authors, without undue reservation, to any qualified researcher.

## Ethics Statement

The study protocol was approved by the Ethics Committee of the Fourth Affiliated Hospital, China Medical University .

## Author Contributions

LS designed, performed the experimental studies, interpreted the data, and drafted the manuscript. XC performed the experimental studies. NX contributed in statistical analysis. HJ designed and edited the manuscript. All authors read and approved the final manuscript.

### Conflict of Interest Statement

The authors declare that the research was conducted in the absence of any commercial or financial relationships that could be construed as a potential conflict of interest.

## Supplementary Material

Supplementary Figure 1 Oncomine data analysis of GnRHR mRNA levels in 16 types of cancer from the Ramaswamy multi-cancer datasets.

## Abbreviations

GnRH: Gonadotropin-releasing hormone
Akt: protein kinase B
ERK: extracellular signal-regulated protein kinase
Bcl-2: B-cell lymphoma-2
Bax: Bcl-2-associated X
LC3: microtubule-associated protein 1 light chain 3
MMP2: matrix metallopeptidase 2
MMP9: matrix metallopeptidase 9.

## References

[1] Siegel RL Miller KD Jemal A Cancer statistics, 2017. CA Cancer J Clin. (2017) 67:7–30. 10.3322/caac.21387 28055103

[2] UICC TNM Classification of Malignant Tumors. 8th ed. Hoboken, NJ: Wiley-Blackwell (2017).

[3] Anthony EL King JC Stopa EG. Immunocytochemical localization of LHRH in the median eminence, infundibular stalk, and neurohypophysis. Evidence for multiple sites of releasing hormone secretion in humans and other mammals. Cell Tissue Res. (1984) 236:5–14. 10.1007/BF00216506 6370455

[4] Stopa EG Koh ET Svendsen CN Rogers WT Schwaber JS King JC. Computer-assisted mapping of immunoreactive mammalian gonadotropin-releasing hormone in adult human basal forebrain and amygdala. Endocrinology. (1991) 128:3199–207. 10.1210/endo-128-6-3199 2036986

[5] Conn PM Crowley WF Jr. Gonadotropin-releasing hormone and its analogs. Annu Rev Med. (1994) 45:391–405. 10.1146/annurev.med.45.1.391 8198390

[6] Grundker C Gunthert AR Westphalen S Emons G. Biology of the gonadotropin-releasing hormone system in gynecological cancers. Eur J Endocrinol. (2002) 146:1–14. 10.1530/eje.0.1460001 11751060

[7] Imai A Ohno T Iida K Fuseya T Furui T Tamaya T. Presence of gonadotropin-releasing hormone receptor and its messenger ribonucleic acid in endometrial carcinoma and endometrium. Gynecol Oncol. (1994) 55:144–8. 10.1006/gyno.1994.1264 7959256

[8] Kakar SS Grizzle WE Neill JD. The nucleotide sequences of human GnRH receptors in breast and ovarian tumors are identical with that found in pituitary. Mol Cell Endocrinol. (1994) 106:145–9. 10.1016/0303-7207(94)90196-1 7534732

[9] Harris N Dutlow C Eidne K Dong KW Roberts J Millar R. Gonadotropin-releasing hormone gene expression in MDA-MB-231 and ZR-75–1 breast carcinoma cell lines. Cancer Res. (1991) 51:2577–81. 2021939

[10] Montagnani Marelli M Moretti RM Mai S Muller O Van Groeninghen JC Limonta P. Novel insights into GnRH receptor activity: role in the control of human glioblastoma cell proliferation. Oncol Rep. (2009) 21:1277–82. 10.3892/or_00000351 19360304

[11] Szepeshazi K Schally AV Keller G Block NL Benten D Halmos G. Receptor-targeted therapy of human experimental urinary bladder cancers with cytotoxic LH-RH analog AN-152 [AEZS-108]. Oncotarget. (2012) 3:686–99. 10.18632/oncotarget.546 22824624 PMC3443252

[12] Lu C Huang T Chen W Lu H. GnRH participates in the self-renewal of A549-derived lung cancer stem-like cells through upregulation of the JNK signaling pathway. Oncol Rep. (2015) 34:244–50. 10.3892/or.2015.3956 25955300

[13] Emons G Grundker C Gunthert AR Westphalen S Kavanagh J Verschraegen C. GnRH antagonists in the treatment of gynecological and breast cancers. Endocr Relat Cancer. (2003) 10:291–9. 10.1677/erc.0.0100291 12790790

[14] Cheung LW Wong AS. Gonadotropin-releasing hormone: GnRH receptor signaling in extrapituitary tissues. FEBS J. (2008) 275:5479–95. 10.1111/j.1742-4658.2008.06677.x 18959738

[15] Montagnani Marelli M Moretti RM Januszkiewicz-Caulier J Motta M Limonta P. Gonadotropin-releasing hormone (GnRH) receptors in tumors: a new rationale for the therapeutical application of GnRH analogs in cancer patients? Curr Cancer Drug Targets. (2006) 6:257–69. 10.2174/156800906776842966 16712461

[16] So WK Cheng JC Poon SL Leung PC. Gonadotropin-releasing hormone and ovarian cancer: a functional and mechanistic overview. FEBS J. (2008) 275:5496–511. 10.1111/j.1742-4658.2008.06679.x 18959739

[17] Hou L Zhang YY Huang Y Yi QY Lv L Zhang TW. Inhibitors of phosphatidylinositol 3'-kinases promote mitotic cell death in HeLa cells. PLoS ONE. (2012) 7:e35665. 10.1371/journal.pone.0035665 22545128 PMC3335795

[18] Hu PF Chen WP Bao JP Wu LD. Paeoniflorin inhibits IL-1β-induced chondrocyte apoptosis by regulating the Bax/Bcl-2/caspase-3 signaling pathway. Mol Med Rep. (2018) 17:6194–200. 10.3892/mmr.2018.8631 29484390

[19] Radziszewska A Schroer SA Choi D Tajmir P Radulovich N Ho JC. Absence of caspase-3 protects pancreatic {beta}-cells from c-Myc-induced apoptosis without leading to tumor formation. J Biol Chem. (2009) 284:10947–56. 10.1074/jbc.M806960200 19213729 PMC2667780

[20] von Alten J Fister S Schulz H Viereck V Frosch KH Emons G. GnRH analogs reduce invasiveness of human breast cancer cells. Breast Cancer Res Treat. (2006) 100:13–21. 10.1007/s10549-006-9222-z 16758121

[21] Dondi D Festuccia C Piccolella M Bologna M Motta M. GnRH agonists and antagonists decrease the metastatic progression of human prostate cancer cell lines by inhibiting the plasminogen activator system. Oncol Rep. (2006) 15:393–400. 10.3892/or.15.2.393 16391860

[22] Annabi B Lachambre MP Plouffe K Sartelet H Beliveau R. Modulation of invasive properties of CD133+ glioblastoma stem cells: a role for MT1-MMP in bioactive lysophospholipid signaling. Mol Carcinog. (2009) 48:910–9. 10.1002/mc.20541 19326372

[23] Zhao L Zhu Z Yao C Huang Y Zhi E Chen H. VEGFC/VEGFR3 signaling regulates mouse spermatogonial cell proliferation via the activation of AKT/MAPK and cyclin D1 pathway and mediates the apoptosis by affecting caspase 3/9 and Bcl-2. Cell Cycle. (2018) 17:225–39. 10.1080/15384101.2017.1407891 29169284 PMC5884123

[24] Li Z Qu L Zhong H Xu K Qiu X Wang E. Low expression of Mig-6 is associated with poor survival outcome in NSCLC and inhibits cell apoptosis via ERK-mediated upregulation of Bcl-2. Oncol Rep. (2014) 31:1707–14. 10.3892/or.2014.3050 24573418

[25] Dondi D Limonta P Moretti RM Marelli MM Garattini E Motta M. Antiproliferative effects of luteinizing hormone-releasing hormone (LHRH) agonists on human androgen-independent prostate cancer cell line DU 145: evidence for an autocrine-inhibitory LHRH loop. Cancer Res. (1994) 54:4091–5. 8033142

[26] Bar-Lev TH Harris D Tomic M Stojilkovic S Blumenfeld Z Brown P. Role of PI4K and PI3K-AKT in ERK1/2 activation by GnRH in the pituitary gonadotropes. Mol Cell Endocrinol. (2015) 415:12–23. 10.1016/j.mce.2015.07.029 26238084 PMC4582010

[27] Schnelldorfer T Ware AL Sarr MG Smyrk TC Zhang L Qin R. Long-term survival after pancreatoduodenectomy for pancreatic adenocarcinoma: is cure possible? Ann Surg. (2008) 247:456–62. 10.1097/SLA.0b013e3181613142 18376190

[28] Grundker C Ernst J Reutter MD Ghadimi BM Emons G. Effective targeted chemotherapy using AEZS-108 (AN-152) for LHRH receptor-positive pancreatic cancers. Oncol Rep. (2011) 26:629–35. 10.3892/or.2011.1340 21667032

[29] Engel JB Schally AV Dietl J Rieger L Honig A. Targeted therapy of breast and gynecological cancers with cytotoxic analogues of peptide hormones. Mol Pharm. (2007) 4:652–8. 10.1021/mp0700514 17705441

[30] Fost C Duwe F Hellriegel M Schweyer S Emons G Grundker C. Targeted chemotherapy for triple-negative breast cancers via LHRH receptor. Oncol Rep. (2011) 25:1481–7. 10.3892/or.2011.1188 21331448

[31] Li D Yang M Liao A Zeng B Liu D Yao Y Linc00483 as ceRNA regulates proliferation and apoptosis through activating MAPKs in gastric cancer. J Cell Mol Med. (2018) 22:3875–86. 10.1111/jcmm.13661 PMC6050491 29761936

[32] Oak C Khalifa AO Isali I Bhaskaran N Walker E Shukla S. Diosmetin suppresses human prostate cancer cell proliferation through the induction of apoptosis and cell cycle arrest. Int J Oncol. (2018) 53:835–43. 10.3892/ijo.2018.4407 29767250 PMC6017185

[33] Fister S Gunthert AR Aicher B Paulini KW Emons G Grundker C. GnRH-II antagonists induce apoptosis in human endometrial, ovarian, and breast cancer cells via activation of stress-induced MAPKs p38 and JNK and proapoptotic protein Bax. Cancer Res. (2009) 69:6473–81. 10.1158/0008-5472.CAN-08-4657 19638591

[34] Fister S Gunthert AR Emons G Grundker C. Gonadotropin-releasing hormone type II antagonists induce apoptotic cell death in human endometrial and ovarian cancer cells *in vitro* and *in vivo*. Cancer Res. (2007) 67:1750–6. 10.1158/0008-5472.CAN-06-3222 17308117

[35] Campbell KJ Tait SWG. Targeting BCL-2 regulated apoptosis in cancer. Open Biol. (2018) 8:180002. 10.1098/rsob.180002 29769323 PMC5990650

[36] Yao Q Chen J Lv Y Wang T Zhang J Fan J. The significance of expression of autophagy-related gene Beclin, Bcl-2, and Bax in breast cancer tissues. Tumour Biol. (2011) 32:1163–71. 10.1007/s13277-011-0219-9 21861179

[37] Yang J Yao S. JNK-Bcl-2/Bcl-xL-Bax/Bak pathway mediates the crosstalk between matrine-induced autophagy and apoptosis via interplay with beclin 1. Int J Mol Sci. (2015) 16:25744–58. 10.3390/ijms161025744 26516844 PMC4632824

[38] Li X Wu D Shen J Zhou M Lu Y. Rapamycin induces autophagy in the melanoma cell line M14 via regulation of the expression levels of Bcl-2 and Bax. Oncol Lett. (2013) 5:167–72. 10.3892/ol.2012.986 23255914 PMC3525347

[39] Kraus S Levy G Hanoch T Naor Z Seger R. Gonadotropin-releasing hormone induces apoptosis of prostate cancer cells: role of c-Jun NH2-terminal kinase, protein kinase B, and extracellular signal-regulated kinase pathways. Cancer Res. (2004) 64:5736–44. 10.1158/0008-5472.CAN-04-1156 15313914

[40] Maudsley S Davidson L Pawson AJ Chan R Lopez de Maturana R Millar RP. Gonadotropin-releasing hormone (GnRH) antagonists promote proapoptotic signaling in peripheral reproductive tumor cells by activating a Galphai-coupling state of the type I GnRH receptor. Cancer Res. (2004) 64:7533–44. 10.1158/0008-5472.CAN-04-1360 15492280

[41] Malik SN Brattain M Ghosh PM Troyer DA Prihoda T Bedolla R. Immunohistochemical demonstration of phospho-Akt in high Gleason grade prostate cancer. Clin Cancer Res. (2002) 8:1168–71. 11948129

[42] Kraus S Naor Z Seger R. Gonadotropin-releasing hormone in apoptosis of prostate cancer cells. Cancer Lett. (2006) 234:109–23. 10.1016/j.canlet.2005.02.038 16546667

[43] Wang M Shu ZJ Wang Y Peng W. Stachydrine hydrochloride inhibits proliferation and induces apoptosis of breast cancer cells via inhibition of Akt and ERK pathways. Am J Transl Res. (2017) 9:1834–44. 28469788 PMC5411931

[44] Liu Y Bi T Wang G Dai W Wu G Qian L. Lupeol inhibits proliferation and induces apoptosis of human pancreatic cancer PCNA-1 cells through AKT/ERK pathways. Naunyn Schmied Arch Pharmacol. (2015) 388:295–304. 10.1007/s00210-014-1071-4 25418891

[45] Zhang L Wang H Zhu J Xu J Ding K Mollugin induces tumor cells apoptosis and autophagy via the PI3K/AKT/mTOR/p70S6K and ERK signaling pathways. Biochem Biophys Res Commun. (2014) 450:247–54. 10.1016/j.bbrc.2014.05.101 24887566

[46] Roy B Pattanaik AK Das J Bhutia SK Behera B Singh P. Role of PI3K/Akt/mTOR and MEK/ERK pathway in concanavalin A induced autophagy in HeLa cells. Chem Biol Interact. (2014) 210:96–102. 10.1016/j.cbi.2014.01.003 24434245

[47] Ba L Gao J Chen Y Qi H Dong C Pan H. Allicin attenuates pathological cardiac hypertrophy by inhibiting autophagy via activation of PI3K/Akt/mTOR and MAPK/ERK/mTOR signalging pathways. Phytomedicine. (2018) 58:152765. 10.1016/j.phymed.2018.11.025 31005720

[48] Huang M Huang B Li G Zeng S. Apatinib affect VEGF-mediated cell proliferation, migration, invasion via blocking VEGFR2/RAF/MEK/ERK and PI3K/AKT pathways in cholangiocarcinoma cell. BMC Gastroenterol. (2018) 18:169. 10.1186/s12876-018-0870-3 30400838 PMC6220519

[49] Li C Rezov V Joensuu E Vartiainen V Ronty M Yin M. Pirfenidone decreases mesothelioma cell proliferation and migration via inhibition of ERK and AKT and regulates mesothelioma tumor microenvironment *in vivo*. Sci Rep. (2018) 8:10070. 10.1038/s41598-018-28297-x 29968778 PMC6030186

[50] Lv B Yang X Lv S Wang L Fan K Shi R. CXCR4 Signaling induced epithelial-mesenchymal transition by PI3K/AKT and ERK pathways in glioblastoma. Mol Neurobiol. (2015) 52:1263–8. 10.1007/s12035-014-8935-y 25326893

[51] Wang Z Qu L Deng B Sun X Wu S Liao J. STYK1 promotes epithelial-mesenchymal transition and tumor metastasis in human hepatocellular carcinoma through MEK/ERK and PI3K/AKT signaling. Sci Rep. (2016) 6:33205. 10.1038/srep33205 27628214 PMC5024114

[52] Chen B Zeng X He Y Wang X Liang Z Liu J. STC2 promotes the epithelial-mesenchymal transition of colorectal cancer cells through AKT-ERK signaling pathways. Oncotarget. (2016) 7:71400–16. 10.18632/oncotarget.12147 27662663 PMC5342087

[53] Cheung LW Leung PC Wong AS. Gonadotropin-releasing hormone promotes ovarian cancer cell invasiveness throughc-Jun NH2-terminal kinase-mediated activation of matrix metalloproteinase (MMP)-2 and MMP-9. Cancer Res. (2006) 66:10902–10. 10.1158/0008-5472.CAN-06-2217 17108127

