# Supplementary figures and images for: The Anti-proliferative Activity of GnRH Through Downregulation of the Akt/ERK Pathways in Pancreatic Cancer
Source: Front Endocrinol (Lausanne). 2019 Jun 17;10:370. doi: 10.3389/fendo.2019.00370 (PMC6590102; doi:10.3389/fendo.2019.00370)

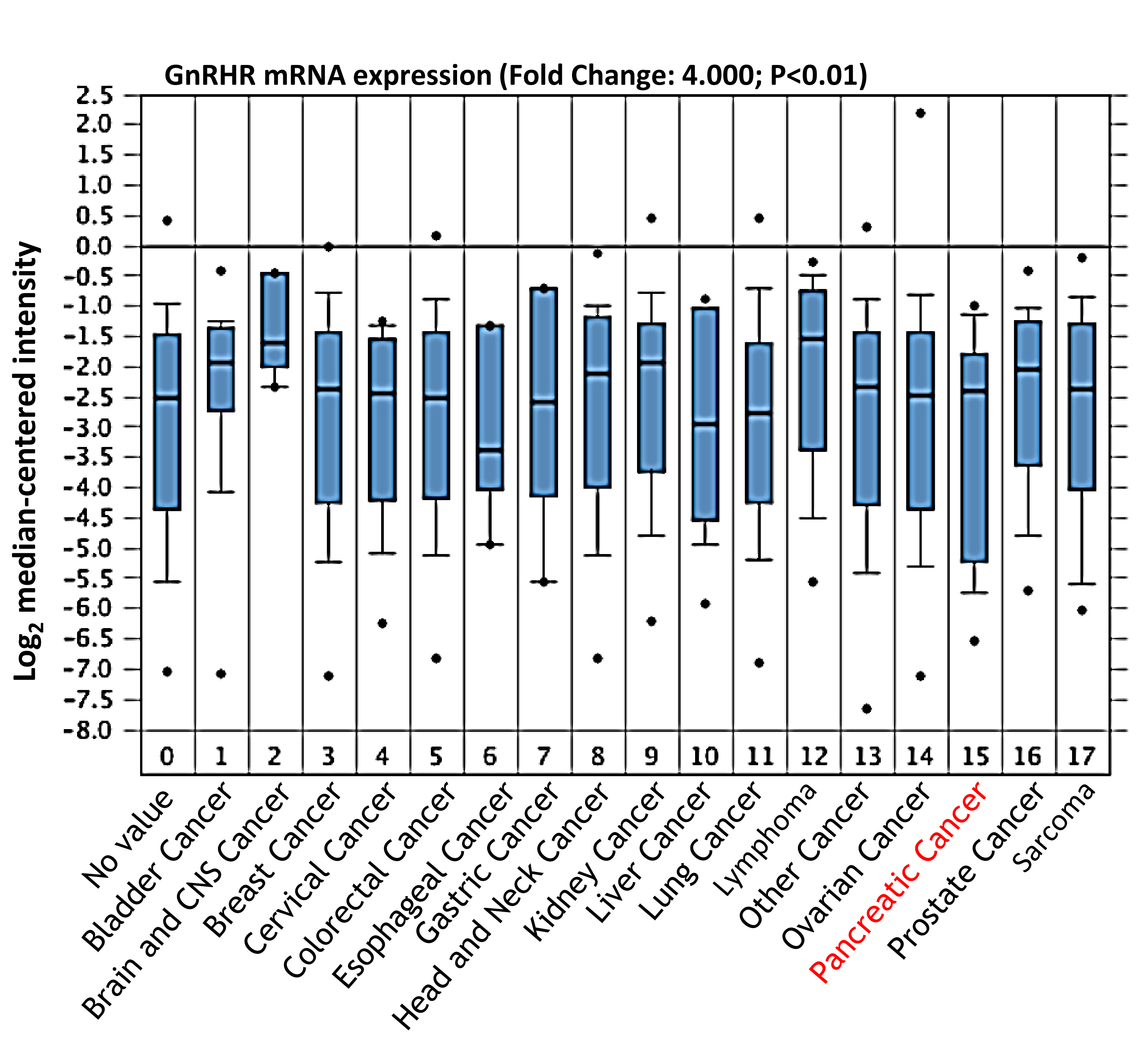

Supplement: Supplementary Figure 1 — Oncomine data analysis of GnRHR mRNA levels in 16 types of cancer from the Ramaswamy multi-cancer datasets. [file Image_1.TIF]
